# Association of tuberculosis and diabetes Mellitus: an analysis of 1000 consecutively admitted cases in a tertiary care hospital of North India

**DOI:** 10.11604/pamj.2016.24.4.8153

**Published:** 2016-05-03

**Authors:** Surinder Pal Singh, Satinder Pal Singh, Jai Kishan, Sumeet Kaur, Shandhra Ramana

**Affiliations:** 1 Department of Tuberculosis and Chest Diseases, Government Medical College, Patiala, Punjab, India; 2 Department of Forensic Medicine and Toxicology, Government Medical College and Hospital, Chandigarh, India; 3 Department of Biochemistry, Government Medical College and Hospital, Chandigarh, India; 4 Department of IST, Penn State University, PA, USA

**Keywords:** Tuberculosis, Diabetes Mellitus, epidemic, death, global health

## Abstract

**Introduction:**

The association of Tuberculosis and Diabetes Mellitus is a cause of concern for the health sector. The coexistence of these two highly prevalent diseases has made the already existing treatments very complex. This issue is of particular significance to developing countries like India that bear a significant burden of these two diseases.

**Methods:**

Retrospective analysis of 1000 consecutively admitted patients in a tertiary care hospital were analyzed for the coexistence of Tuberculosis and Diabetes Mellitus.

**Results:**

The study found that a significant proportion of diabetic patients had coexistent tuberculosis (65.5%). Rural population was predominantly affected in both the genders. The study observed that the coexistence of these two conditions increased with advanced age.

**Conclusion:**

The coexistence of Diabetes Mellitus with Tuberculosis needs to identified early and adequately addressed. The rural population needs to be educated about these two conditions and seek timely medical care.

## Introduction

Tuberculosis and Diabetes Mellitus are the two main diseases that are widely prevalent in India. The convergence of these two conditions has led to increased rates of prolonged treatment, relapse and death. The Tuberculosis- Diabetes Mellitus (TB-DM) epidemic is challenge for India and other developing countries that constitute a major global burden of these two diseases. More than 95% of the total deaths from Tuberculosis worldwide occur in low and middle income group countries. India reports more than two lakh deaths annually from Tuberculosis while the African continent alone reported more than 3,90,000 deaths related to this disease in the year 2009 [1]. With the implementation of Revised National Tuberculosis Control Programme (RNTCP), India has been able to reduce the prevalence of Tuberculosis to a great extent. According to a WHO report, the prevalence of Tuberculosis in India has reduced from 465 per 100,000 in 1990 to 230 per 100,000 in 2012 [2]. Still India reported around 2.3 million cases of Tuberculosis in 2013 of a total of 9 million cases that occurred worldwide, thus constituted one fourth of the annual global burden of the disease [3]. On the other hand, the prevalence of Diabetes Mellitus is increasing globally. In the year 2013, there were more than 380 million diabetic patients in the world. Its prevalence is likely to cross 590 million by 2030. A vast majority of diabetics (more than 80%) belong to low-and middle-income countries and a majority of the patients are in their 5th and 6th decade of their lives. During 2013, there were around 65 million diabetic patients in our country and the number is likely to reach 109 million by 2035 [4–5]. The WHO reports that 11% urban and 3% of rural population in India above the age of 15 years have Diabetes Mellitus.

### The tuberculosis-diabetes Mellitus epidemic

It is known that diabetics have a greater susceptibility to develop Tuberculosis as compared to non- diabetics. It is believed that the impairment of immunity in long term diabetics makes the patients prone to a number of microbial infections including Mycobacterium Tuberculosis. The hyperglycemia in Diabetes Mellitus is believed to favor the growth of the Tuberculosis bacilli. The association between Tuberculosis and Diabetes Mellitus is bidirectional. One of the first line anti tubercular drugs, Rifampicin, is known to interfere with the metabolism of oral hypoglycemic agents and hence affect glycaemic control [6]. The Diabetes Mellitus increases the chance of adverse treatment outcome, relapse and death in patients of Tuberculosis [7–9]. The patients with these two conditions coexisting are more likely to be sputum positive [10] and usually require longer time to be rendered sputum negative. In addition, such patients also have a much higher risk (about 4 times) of death during treatment of Tuberculosis. In our country, a substantial number of Tuberculosis patients have Diabetes Mellitus [11]. As the number of diabetics is rising worldwide, there are greater chances that Diabetes Mellitus will interfere with control and care of Tuberculosis [10, 12]. The convergence of Diabetes Mellitus and Tuberculosis has increased the interest to study various aspects of human immunity in relation to Tuberculosis that can be helpful to provide better care to the general public.

## Methods

The present retrospective study was conducted at Department of Tuberculosis and Chest Diseases, Government Medical College, Patiala, Punjab, India. A total of 1000 consecutively admitted cases for various respiratory diseases from April 2013 to August 2013 were subjected to detection of Tuberculosis and Diabetes Mellitus. Only those patients in whom these two conditions were confirmed to coexist were included in the study. The cases in which only one of the two conditions was present were excluded from the study. The detection of Tuberculosis was based on history, signs & symptoms (i.e. fever, cough, expectoration, loss of weight, loss of appetite, fatigue etc) and investigations (including Chest X rays, sputum examination for Acid Fast Bacilli, bronchoscopy etc). Then the diagnosed patients of Tuberculosis were asked of history of Diabetes Mellitus as these two conditions are known to coexist in the same individual. The patients of tuberculosis patients who confirmed their Diabetes Mellitus (i.e. already on medications for Diabetes mellitus) were included as such in the study. Those patients who did not know about their Diabetes Mellitus status were subjected to fasting blood glucose examinations. They were then either included or excluded from the study based on the presence or absence of Diabetes Mellitus. The patients of all age groups and both genders were included in the study. The patients with other medical conditions like COPD, asthma, heart diseases, HIV, long term use of corticosteroids, malignancies etc were excluded from the study.

## Results

Of a total of 1000 cases studied, the coexistence of tuberculosis with diabetes mellitus was found only in 76 cases. Out of these, 61 had pulmonary tuberculosis and 15 had extra pulmonary tuberculosis. All these cases were included in the study. Thus, only 76 cases out of a total of 1000 cases fulfilled the inclusion criteria. The study population included only the confirmed cases of tuberculosis. In 544 cases (54.4%) only tuberculosis (but not Diabetes mellitus) was present and therefore excluded from the study. There were 340 patients who were admitted with respiratory problems other than tuberculosis and hence also excluded from the study. The diabetes mellitus in association with other respiratory problems (but not tuberculosis) was present in 40 cases and hence excluded from the study. The males constituted a majority of cases in both Tuberculosis-Diabetes Mellitus group (56.5%) and in Tuberculosis only group (60.3% cases). The mean age of Tuberculosis-Diabetes Mellitus patients was found to be 56.67± 12.24 years. The mean age of Tuberculosis only patients was 41.60 years ±18.66 years. The maximum number of cases of Tuberculosis-Diabetes Mellitus belonged to 51-60 years age group (35.5%) followed by above 60 years age group (30.1%). No case of Tuberculosis-Diabetes Mellitus was reported below the age of 30 years. In contrast, the maximum number of cases of Tuberculosis only group belonged to 21-30 years age group (21.3%) followed equally by 41-50 year age group and above 60 years age groups (16% each). The least number of cases belonged to 0-10 year's age group (0.9%) (Table 1). The present study observed that most of the Tuberculosis-Diabetes Mellitus patients belonged to rural areas (68.4%). Similar results were also obtained in Tuberculosis only group (72.2%) (Table 2). The examination of radiological investigations did not reveal any significant differences between the two groups (Table 3).

**Table 1:** Spread of cases among different age groups

Age group	No. of cases of coexisting Tuberculosis-Diabetes Mellitus	No. of Tuberculosis cases without coexisting Diabetes Mellitus
≤ 10 years	-	5
11-20	-	78
21-30	-	116
31-40	7	88
41-50	19	87
51-60	27	83
> 60 years	23	87
Total	76	544

**Table 2:** Spread of cases among rural and urban population

Area of residence	No. of cases of coexisting Tuberculosis-Diabetes Mellitus	No. of Tuberculosis cases without coexisting Diabetes Mellitus
Rural	52	393
Urban	24	151
Total	76	544

**Table 3:** The radiological findings of the cases

Parameter		Diagnosis			
		Tuberculosis-Diabetes Mellitus		Tuberculosis only	
		No. of cases	Percent	No. of cases	Percent
Lungs	Left Lung	8	10.5%	89	16.3%
	Right Lung	16	21.1%	108	19.9%
	Both Lungs	20	26.3%	131	24.0%
	Not involved	32	42.1%	216	39.8%
Cavitatory lesions	Absent	70	92.1%	522	95.9%
	Present	6	07.9%	22	04.1%
Exudative lesions	Absent	65	85.5%	470	86.4%
	Present	11	14.5%	74	13.6%
Infiltrative lesions	Absent	46	60.5%	297	54.6%
	Present	30	39.5%	247	45.4%
Pleural effusion	Absent	75	98.7%	539	99.1%
	Present	1	01.3%	5	0.9%
Total no. of cases	-	76	-	544	-

## Discussion

The present study retrospectively analyzed 1000 consecutively admitted cases to a leading Tuberculosis and chest hospital of north India for the coexistence of Tuberculosis and Diabetes Mellitus. These cases constituted 11.6% of the total admitted cases during the study period. The present study observed that males were the main victims in a majority of cases. Similar findings have been reported from other parts of India, Japan and Ethiopia [13–17]. Contrasting results (female preponderance) were reported from Iran [18]. The poor treatment results in males might be because of their non-adherence to treatment [19–21]. The male predominance can possibly be explained by their more frequent exposure to Tuberculosis patients due to greater involvement in outdoor activities. A number of studies have observed that Diabetes Mellitus is also more commonly associated with males. The greater stresses of life have also been observed in males and it could lead to overeating making them more susceptible to fluctuations in blood sugar levels. The mean age in the Tuberculosis-Diabetes Mellitus patients in the present study was found to be 56.67± 12.24 years while in cases of Tuberculosis only, it was 41.60 ± 18.66 years. Similar results were reported from Iran [22] and Ethiopia [17]. It has been reported that age above 46 years is associated with poor treatment results [23]. The decreased immunity and general decline of physical health and lesser ability to reach the health care centers that occur with advancing age might be the reasons for poor results in advanced ages [24–25]. In the age of 40 to 60 years, increased involvement with work related activities might be associated with poor adherence with treatment [20]. Most of the cases in the present study belonged to rural area. Concordant findings were found in Malaysia [20]. However, this study is in disagreement with other Indian studies that reported greater involvement of urban population in both Tuberculosis and Diabetes Mellitus as it is associated with increased crowding, sedentary life and urbanization [26]. Delay in seeking medical care and poor compliance with treatment might have contributed to greater incidence among the rural population in this region of the country. No statistical significance was observed among the distribution of different types of lesions in the lungs.

## Conclusion

The present study concluded that early screening for Diabetes Mellitus in Tuberculosis patients is likely to increase the detection of Diabetes Mellitus. This will lead to an early recognition of the coexistence of these two conditions and therefore appropriate measures could be taken to simultaneously treat these conditions. Though RNTCP programme has been quite effective in India, it can be further improved to monitor and report such a coexistence of Tuberculosis with other conditions like Diabetes Mellitus and achieve a further fall in morbidity and mortality.

### What is known about this topic

- Diabetes is important risk factor for Tuberculosis and might affect its presentation and treatment and outcome;
- Tuberculosis induces glucose intolerance and worsens the glycaemic control in Diabetic patients;
- Poorly controlled DM leads to multiple complications and increased susceptibility to infections. Diabetes leads to increased susceptibility to Tuberculosis via different mechanisms. One mechanism include those directly related to hyperglycemia and cellular insulinopenia, and other is effects on macrophage and lymphocyte function, leading to diminished ability to contain the organism. Altered immunity in DM leads to increased microbial burden which lead to longer time of conversion and higher rate of relapse.

### What this study adds

- In the area of present study, there is a large population of diabetics and tuberculosis patients and there is increasing number of cases of multidrug resistance tuberculosis. The poorly controlled DM is an important factor that contributes towards drug resistance;
- We reviewed epidemiology of TB and DM and provided a synopsis of the evidence for the role of Diabetes mellitus in susceptibility to, clinical presentation of, and response to treatment for tuberculosis;
- There are only a few studies that have been conducted in this area of India and hence more research is essential to counter the coexistence of these two medical conditions

## References

[1] World Health Organization (2010). http://reliefweb.int/sites/reliefweb.int/files/resources/F530290AD0279399C12577D8003E9D65-Full_Report.pdf.

[2] India. Directorate General of Health Services (2014). Ministry of Health and Family Welfare, Nirman Bhavan.

[3] World Health Organization (2014). WHO Global Tuberculosis Report.

[4] International Diabetes Federation (2013). IDF Diabetes Atlas.

[5] Lee MY, Lin KD, Hsu WH, Chang HL, Yang YH, Hsiao PJ, Shin SJ (2015). Statin, calcium channel blocker and beta blocker therapy may decrease the incidence of Tuberculosis infection in elderly Taiwanese patients with type 2 Diabetes. Int J Mol Sci..

[6] Wadhwa N (2015). Tuberculosis and Diabetes - The converging epidemic. J Clin Prev Cardiol..

[7] Dooley KE, Chaisson RE (2009). Tuberculosis and Diabetes Mellitus: convergence of two epidemics. Lancet Infect Dis..

[8] Baker MA, Harries AD, Jeon CY, Hart JE, Kapur A, Lonnroth K, Ottmani SE, Goonesekera SD, Murray MB (2011). The impact of diabetes on tuberculosis treatment outcomes: a systematic review. BMC Med..

[9] Jimenez-Corona ME, Cruz-Hervert LP, Garcia-Garcia L, Ferreyra-Reyes L, Delgado-Sanchez G, Bobadilla-Del-Valle M (2013). Association of diabetes and tuberculosis: impact on treatment and post-treatment outcomes. Thorax..

[10] Stevenson CR, Forouhi NG, Roglic G, Williams BG, Lauer JA, Dye C, Unwin N (2007). Diabetes and Tuberculosis: the impact of the diabetes epidemic on tuberculosis incidence. BMC Public Health..

[11] Viswanathan V, Kumpatla S, Aravindalochanan V, Rajan R, Chinnasamy C, Srinivasan R, Selvam JM, Kapur A (2012). Prevalence of diabetes and pre-diabetes and associated risk factors among Tuberculosis patients in India. PLoS One..

[12] Tuberculosis & diabetes: the growing threat of the double burden of Diabetes and Tuberculosis. International Union Against Diabetes Mellitus and Lung Diseases.

[13] Guptan A, Shah A (2000). Tuberculosis and Diabetes: An appraisal. Ind J Tub..

[14] Deshmukh PA, Shaw T (1984). Pulmonary tuberculosis and diabetes mellitus. Ind J Tub..

[15] Kishan J, Garg K (2010). Tuberculosis and Diabetes Mellitus: A case series of 100 patients. SAARC J Tuber Lung Dis HIV/AIDS..

[16] Yamagishi F, Suzuki K, Sasaki Y, Saitoh M, Izumizaki M, Koizumi K (1996). Prevalence of coexisting diabetes mellitus among patients with active pulmonary tuberculosis. Kekkaku..

[17] Amare H, Gelaw A, Anagaw B, Gelaw B (2013). Smear positive pulmonary tuberculosis among diabetic patients at the Dessie referral hospital, Northeast Ethiopia. Infect Dis Poverty..

[18] Baghaei P, Tabarsi P, Abrishami Z, Mirsaeid M, Faghani YA, Mansouri SD, Masjedi MR (2010). Comparison of pulmonary TB patients with and without diabetes mellitus Type II. TANAFFOS..

[19] Balasubramanian R, Garg R, Santha T, Gopi PG, Subramani R, Chandrasekaran V, Thomas A, Rajeswari R, Anandakrishnan S, Perumal M, Niruparani C, Sudha G, Jaggarajamma K, Frieden TR, Narayanan PR (2004). Gender disparities in tuberculosis: report from a rural DOTS programme in south India. Int J Tuberc Lung Dis..

[20] Sulaiman SA, Khan AH, Muttalif AR, Hassali MA, Ahmad N, Iqubal MS (2013). Impact of Diabetes mellitus on treatment outcomes of Tuberculosis patients intertiary care setup. Am J Med Sci..

[21] Feng JY, Huang SF, Ting WY, Chen YC, Lin YY, Huang RM, Lin CH, Hwang JJ, Lee JJ, Yu MC, Yu KW, Lee YC, Su WJ (2012). Gender differences in treatment outcomes of tuberculosis patients in Taiwan: a prospective observational study. Clin Microbiol Infect.

[22] Alavi SM, Khoshkhoy MM (2012). Pulmonary tuberculosis and diabetes mellitus: co-existence of both diseases in patients admitted in a teaching hospital in the southwest of Iran. Caspian J Intern Med..

[23] Talay F, Kumbetli S, Altin S (2008). Factors associated with treatment success for Tuberculosis patients: a single center's experience in Turkey. Jpn J Infect Dis..

[24] Berhe G, Enquselassie F, Aseffa A (2012). Treatment outcome of smear positive pulmonary tuberculosis patients in Tigray region, northern Ethiopia. BMC Public Health..

[25] Munoz-Sellart M, Cuevas LE, Tumoto M, Merid Y, Yassin MA (2010). Factors associated with poor tuberculosis treatment outcome in the southern region of Ethiopia. Int J Tuberc Lung Dis..

[26] Sen T, Joshi SR, Udwadia ZF (2009). Tuberculosis and diabetes mellitus: merging epidemics. JAPI.

